# SOSORT 2012 consensus paper: reducing x-ray exposure in pediatric patients with scoliosis

**DOI:** 10.1186/1748-7161-9-4

**Published:** 2014-04-25

**Authors:** Patrick Knott, Eden Pappo, Michelle Cameron, JC deMauroy, Charles Rivard, Tomasz Kotwicki, Fabio Zaina, James Wynne, Luke Stikeleather, Josette Bettany-Saltikov, Theodoros B Grivas, Jacek Durmala, Toru Maruyama, Stefano Negrini, Joseph P O’Brien, Manuel Rigo

**Affiliations:** 1 The 2012 SOSORT Conference, Milan, Italy

## Abstract

This 2012 Consensus paper reviews the literature on side effects of x-ray exposure in the pediatric population as it relates to scoliosis evaluation and treatment. Alternative methods of spinal assessment and imaging are reviewed, and strategies for reducing the number of radiographs are developed. Using the Delphi technique, SOSORT members developed consensus statements that describe how often radiographs should be taken in each of the pediatric and adolescent sub-populations.

## The epidemiology of x-ray exposure in pediatric patients with spinal deformity

Scoliosis is a relatively common disorder with pathologic spinal curves greater than 20° occurring in approximately 2-4% of children aged six to fourteen [1]. To date, the gold standard for identifying and monitoring scoliosis has been standing anteroposterior (AP) and lateral scoliosis x-ray films, with systematic radiographic imaging performed throughout the individual’s course of treatment. As such, significant advances have been made in current diagnostic techniques: in general, adolescents are exposed to less radiographic imaging, and newer imaging techniques (e.g., 3-phase x-ray machines and high-speed x-ray films) decrease radiation exposure. It should be noted that the rates of cancer secondary to radiation exposure are not specific to scoliosis patients or x-rays. Numerous studies have documented the increase in ionizing radiation exposure and the corresponding risk of cancer in individuals with tuberculosis who require frequent lung radiographs to ensure infection progression, as well as patients who were exposed to atomic bomb radiation [2].

However, there is an increasing awareness of the potential oncogenic effect of radiation exposure. Ronckers et al. studied patients diagnosed with scoliosis between 1912 and 1965 who were exposed to significant ionizing radiation in their adolescent years [3]. The median value for cumulative dose for the breast alone was 10–15 cGy (centi-Gray radiation unit, where one Gray is the absorption of one Joule of energy of ionizing radiation). In a child, neuropsychiatric damage is possible at 1,800 cGy, and endocrinologic dysfunction of the pituitary gland is seen at approximately 2,000 cGy [4]. Ronckers et al. followed 5,513 females who were exposed to an average of 22.9 radiographs per person during treatment and follow-up of scoliosis. Overall, the risk of mortality was 46% higher than the general population, with cancer identified as the primary cause of 23% of these deaths. In terms of frequency, breast cancer was most common, followed by lung and then ovarian cancer. Surprisingly, an increased risk was not identified in terms of developing thyroid cancer or leukemia, both of which were predicted to have increased risk secondary to radiation exposure.

A key aspect of this study was that the risk of death secondary to breast cancer corresponded with the number of x-rays involving breast radiation exposure. It was found that women with 25–49 x-rays involving breast exposure were 1.4 times more likely to die of breast cancer than women with fewer than 25 x-rays, and women with more than 50 x-rays were 2.7 times more likely to die of breast cancer. In addition, the number of xrays paralleled the amount of actual radiation exposure, with an increased rate of breast cancer in women exposed to higher than 20 cGy of radiation compared to women exposed to 0–9 cGy of radiation. These findings were not replicated in the analyses of the relationship between radiation and lung cancer. One possible explanation is that the lungs are exposed to only a fraction of the radiation doses that breast tissue is exposed to. The study also noted a significantly decreased risk of cervical cancer, which is likely due to the fact that some females with scoliosis suffer from sexual dysfunction. Several specific aspects of the group followed in Ronckers et al. are noteworthy: women with scoliosis smoked slightly less than the general public but included the same number of heavy smokers, and had higher rates of sexual dysfunction (thus reducing HPV transmission and cervical cancer risk while increasing breast cancer cases because nulliparous women are at higher risk). Additionally, if women with scoliosis are not physically able to withstand aggressive cancer treatment, the numbers associated with mortality may be skewed. In summary, the relationship between radiation dosage and cancer itself is a key element in the study that supports a correlation between breast cancer and radiation exposure.

Another key study by Nash et al. followed 13 females with idiopathic adolescent scoliosis [5]. AP and lateral films were measured over a 3-year period, during which the females were part of a treatment program for back brace curvature reduction. This Milwaukee-based program estimated that each patient had 22 films taken during the 3-year course. The study showed that the increased risk for leukemia was 3.4%, stomach and upper gastrointestinal cancers was 1.3%, lung cancer was 7.5%, and breast cancer secondary to the radiation exposure was 110%. This risk is reduced to only 3.8% if posteroanterior (PA) films are taken rather than AP films. The fact that these numbers were so drastically altered by adjusting the mechanics of the radiographic imaging led to the study’s recommendation to use PA films rather than AP films. Gialousis et al. also found that the PA approach could reduce the increased risk of breast cancer in females treated for scoliosis [6].

A study by Levy et al., which concluded that the effects of radiation exposure depend on certain variables [7], looked at 2,039 patients who were at least 9 years old between 1965 and 1979. The study found that, for several different types of full-spinal radiographs, the doses of radiation were 50% lower for adolescents compared to adults. This reduced dose is due to increased intensity and energy of the x-ray to compensate for the larger body size of adult patients. In an AP view, the thyroid gland and breast were most exposed, but the PA view nearly eliminated thyroid radiation exposure. It should be noted that the PA view did increase exposure to lungs in women and bone marrow in both sexes; however, both of these malignancies are known to have less risk per dosage of radiation exposure. Further, the study explains that both severity of the scoliotic curve and whether the patient had surgery are important factors that increased radiation exposure in this specific cohort. In essence, more severe curvatures require more images to track the progression of the curve. If the patient was older when diagnosed, the number of radiographs was reduced because they were not exposed to as much monitoring via imaging. Overall, the mean number of radiographs taken was 12 for females and 10 for males. For females, patients diagnosed under the age of 13 who had curves less than 20° had a lifetime cancer risk of 65 per 100,000. Females under the age of 13 who had surgical intervention for the curvature had an overall lifetime risk of cancer of 238 per 100,000. The numbers were less drastic for men, as the incidence of breast cancer was clearly negligible. Again, as seen in Nash et al., the greatest increased risk of any one particular cancer is breast cancer, and substituting AP imaging with PA imaging again reduces the risk. It should be noted that the study did find an increase in females having gastrointestinal malignancies from the PA angle imaging technique.

After following 5,573 patients who had diagnostic testing for scoliosis, Doody et al. also showed that the risk of breast cancer in females is increased. This study also pointed out the importance of age and that females who were exposed between the ages of 10–11 had a greater risk of cancer with a greater dose–response relationship when compared to females exposed at an older age [8].

A review of these studies creates some consistent conclusions, despite varying calculations of risks of cancer and assumptions of radiation exposure per x-ray and over a lifetime. First, PA films should replace AP films, when possible, to reduce excess radiation to both the thyroid and breast tissue. This specific positioning is most important in females because of the specific increased risk of breast cancer from the radiation exposure. Second, while many of the original studies discussing the harmful effects of radiation produced dramatic results, they were using outdated techniques of radiation; current diagnostic procedures are less damaging than previously thought. Third, the earlier the exposure in a patient’s life, the more harmful; therefore, delayed imaging may be of benefit in terms of radiation exposure. Delayed imaging may, however, hinder the ultimate treatment of the scoliosis. Finally, while there is an overall increased risk of cancer, if the images are taken as infrequently as possible and if positioning encourages the safety of the patient, the risks to the patient can be minimized. Imaging should be done as necessary to provide the best patient care possible, balancing both risks and benefits. Any disparities in calculations or risk calculations are likely due to the lack of standard measurement of radiation as well as differences for each x-ray machine and the positioning of every individual patient.

## Use of the Bunnel scoliometer as an indicator of deformity

The scoliometer was described by WP Bunnell in 1984 as a simple, reliable, inexpensive measurement of trunk asymmetry [9]. This trunk asymmetry, which is caused by the rotation and deformity of the rib cage, is related to the magnitude of the scoliosis curve. However, the scoliometer does not directly measure scoliosis, which is traditionally assessed using the radiographs and measured by Cobb angle. The accuracy (inter and intra reliability) of the scoliometer was previously reported by Murrell et al. in 1993 [10]. The method used for the reliability study and the results was reported in the article by Grivas et al. in 2006 [11].

Murrell et al. also reported on the reliability of the scoliometer, noting that there was near perfect agreement between the thoracic scoliometer measurements in degrees (with an intrareader error of 1.2°) and lumbar scoliometer measurements in degrees (with an intrareader error of 1.6°). They concluded that the scoliometer can be used reliably by a single trained observer to measure trunk rotation, and noted that the authors’ current practice is to use the scoliometer as part of every physical examination of patients being screened for, or with, scoliosis [10].

In 1990, Amendt et al. reported that scoliometer measurements made by two raters on 65 individuals with idiopathic scoliosis were correlated with radiographic agreement of vertebral (pedicle) rotation and lateral curvature (Cobb method) [12]. Correlations ranged from .32 to .46 with pedicle rotation, and from .46 to .54 with the Cobb angle. Frequency analysis revealed relatively good specificity, sensitivity, and predictive capability of the scoliometer. Intrarater and interrater reliability coefficients were high (r = .86-.97). These results indicate good measurement reproducibility. The less-than-optimal between-method correlation coefficients suggest that the validity of scoliometer measurements is not sufficient to use this method alone for determining patient diagnosis and management. Based on the positive-frequency analysis, however, the use of this tool as a screening device would be appropriate.

These findings can be accepted, especially if we consider new knowledge based on recently published research on the correlation of surface (trunkal) and axial (spinal) deformity by Grivas et al. in 2007 [13]. This research documented that growth has a significant effect in the correlation between the thoracic and spinal deformity in girls with idiopathic scoliosis. In younger children, the concordance of the surface and spinal deformity is weak, but becomes stronger as the children grow. Therefore, in younger children with surface/trunk asymmetry, the prediction of the spinal deformity alone from the surface topography is inaccurate. Consequently, this knowledge should be taken into consideration when assessing spinal deformity based on surface measurements and correlating surface and radiological readings.

In 1988, Huang also reported on the effectiveness of this instrument by studying the correlation of scoliometer with radiographical readings [14]. He concluded that the value of the scoliometer in school scoliosis screening needs further evaluation, which, in our opinion, underestimates the value of the scoliometer for screening asymmetry and is, therefore, not acceptable. The age range of screened children by Huang et al. was 12–14 years old. As we discuss below, in this age range of screened children the correlation of surface and radiographical deformity is not statistically significant; therefore, the author’s findings were expected and predicted. In addition, as stated by Bunnell et al. and others, rib asymmetry does not always equate to vertebral column asymmetry [9].

As previously reported, in children younger than the age of 14 who have double rib contour sign (DRCS) and rib hump (rib index > 1), the correlation of surface (rib index) and radiographical (Cobb angle) deformity is not statistically significant, which is the case in children older than the age of 14 [13].

Analyzing the above statement, it is useful to say that all lateral spinal radiographs in idiopathic scoliosis show a DRCS of the rib cage, a radiographic expression of the rib hump. The outline of the convex overlies the contour of the concave ribs [15]. The DRCS results primarily from rib deformation and secondarily from vertebral rotation because DRCSs could be present in straight spines with no vertebral rotation. In all our school-screening referrals (having ATI > 7°), the thorax deformity, in terms of the DRCS/hump, has already been developed, and 70% of these children were scoliotics. The rest had a curvature of less than 9° of Cobb angle (10%) or they were children with straight spines (20%) who were followed due to the existing rib hump. The non-scoliotics were 1.5 - 2 years younger than the ones who had already developed scoliosis, and they had an approximate “rib index” of 1.5. The DRCS is present in all referrals, as the DRCS is always present in scoliotic lateral spinal radiographs with no exception [13]. This observation supports the hypothesis that, in idiopathic scoliosis, the deformity of the thorax develops first and the deformity of the spine succeeds.

## The history of surface topography in the prediction of spinal deformity

Scoliosis is a relatively common disorder, with curves greater than 20° occurring in <1/1000 [1]. Radiographs are the current gold standard for identifying and monitoring scoliosis but they have a number of disadvantages, including 5° and 6.5° intra- and inter-observer variation, respectively, in Cobb angle measurements [16]. Another disadvantage is the potential oncogenic effects of radiation exposure, specifically breast cancer, the risk of which has been found to be 4 to 10 times higher in females with scoliosis [3,17]. This risk includes increased rates of overall cancer mortality as high as 8% [3] and a 4.1 times higher mortality rate from breast cancer in woman receiving 50 or more radiographs with a lag time of 30 years [18,19].

Because of these disadvantages and significant potential risks associated with radiographs, there have been numerous attempts to find alternative methods to diagnose and monitor scoliosis, and surface topography has become an increasingly viable option for both. As one of the earliest forms of surface topography, Moiré technology is based on the distortion that occurs when a grid is projected onto a 3D object; the changes in the grid are then used to extrapolate the contour of the surface [20]. Moiré was first developed in the 1960-70s and reported on separately by Takasaki [21] and Meadows et al. [22] in 1970. Subsequently in the 1980s and 90s, researchers began using it to assess back surface topography to determine the curvature of the spine. Early analysis was incredibly burdensome, required hours to process, and was highly influenced by excess noise.

In the 1980s, Drerup and Hierholzer developed new technologies based on Raster stereography, which was similar to Moiré technology but Raster projected narrow black and white stripes of light rather than a grid onto the patient’s trunk and, like Moiré, its distortion was used to extrapolate the curvature of the spine [23]. Raster’s adoption resulted in Moiré gradually being phased out.

Several different systems currently use Raster technology, the most common being InSpeck, ISIS, Quantec, and Frometric. Each version has a slightly different term for their version of the Cobb angle, but all four have been found to be highly reproducible and correlate well with radiographs. InSpeck uses two or four optical digitizers and a structured light projector, while the other systems use only one.

InSpeck was developed in 2002 and takes five data sets in 4–6 seconds. Pazos et al. found that InSpeck provided reproducible and accurate results for both the anatomic and clavicle positions [24]. Seoud et al. used it in combination with radiographs to create a 3D geometric model of the rib cage and found an accuracy of 1.1 ± 0.9 mm over the entire trunk surface, with a 1.4° surface rotation error [25]. Fortin et al. used it with a pressure mat to develop braces [26]. Labelle et al. found a statistically significant improvement in curve correction when using InSpeck to develop their braces compared to the control group [27].

Integrated Shape Imaging System (ISIS1) was developed between 1984 and 1988, with a scan time of 2 seconds, and required 10 minutes to analyze a photograph. The second version, ISIS2, had a scan time of <0.1 second and a fringe frequency of 0.16 fringes/mm, with an accuracy of +/- 1 mm. Zubovic et al. performed 520 scans and found good repeatability and no statistically significant differences when compared to radiographic measurements [28]. Berryman et al. examined patients using a positioning system. Their version of the Cobb angle is lateral asymmetry, which they found to have good correlation (R = 0.84) within 10° of the Cobb angle in 80% of patients [29]. They also used it to measure rib hump height, and found the difference between paired measurements to be -0.08 mm plus or minus <1 cm [30]. They then measured thoracic kyphosis and found an average kyphosis angle of 33.8°, with the mean difference between pairs of measurements totaling -0.02° +/- 7.4° 95% CI [31]. This compares to radiographs, where Carmen et al. found a +/-10.6° 95% CI inter-observer variation and a +/-10.4° 95% CI intra-observer variation [32].

The Quantec system is highly portable, takes 250,000 data points with an accuracy of 0.25 mm in only 1/50th of a second, and has been used by McArdle and others since 1994 [33-35]. McArdle et al. measured thoracic sagittal curve on five or more occasions prior to surgery and found an average standard deviation of 3.8° [36]. Klos et al. used a positioning device and found their intraday Q angle (their equivalent of the Cobb angle) variation was <5°. However, they found that the Q angle may not be sufficient to monitor the Cobb angle because when the Q angle increased <5°, the Cobb angle varied more, but when the Q angle increased >5°, the Cobb angle varied less [37]. McDonald et al. used the Q angle to analyze the effects of maximum sway by having the patient stand entirely on one foot, then the other, and compared that to their baseline of standing equally on both feet. They found that the thoracic curve and pelvic tilt measurements were most profoundly affected and that the changes in all the measures were small except for pelvic tilt [38].

The Formetric system acquires 12 data sets in 6 seconds. In Knott et al.’s reproducibility study, they found that the scoliosis angle of the major curve (equivalent to the Cobb angle) had an average standard deviation of +/- 3.2° [39,40]. Hackenberg et al. used it to compare axial rotation standing versus forward flexed and found a 3.2° increase in back surface rotation between the two postures with a standard deviation of 6.1°, and a poor correlation between the axial rotation in standing and forward bending positions measured with both surface topography (r^2^ = 0.41) and scoliometer (r^2^ = 0.35) [41]. Mohokum et al.’s reliability study examined the influence of BMI above and below 24.99 and found BMI did not affect reproducibility [42]. Contrarily, Knott et al. did find increased variability in scoliosis angles at greater BMIs; however, even at the highest BMIs, the variability in their scoliosis angle measurements was only +/- 4.6° [43]. ISIS2’s lateral asymmetry was also limited in patients who were extremely obese or very muscular [29].

### Conclusions on topography

There are currently several different systems that measure surface topography, and current data supports the accuracy and reproducibility of all four systems. Multiple studies have demonstrated that, while their Cobb angle equivalents may not be an exact match, they approximate it well and their changes parallel changes in the Cobb angle. These approximations make it feasible to use surface topography to both screen and follow patients with scoliosis, only obtaining radiographs when there has been a change in their surface topography measurements. Although surface topography has not completely eliminated the need for radiographs, as an x-ray is still needed to evaluate the morphology of the spine, it has the potential to dramatically decrease the number of radiographs taken over the lifespan of the patient.

Future areas of research include validating the use of surface topography in larger patients and the use of posturing devices, especially in the neuromuscular population, and surface markers, especially in the obese and neuromuscular population.

## The use of low dose x-ray imaging in spinal deformity

Attempts to reduce x-ray exposure in patients has led researchers to develop imaging machines that use very low doses of radiation. Slit scan technology, such as that used by EOS Imaging, allows a spinal image to be taken in two projections simultaneously, using only a fraction of the radiation exposure of standard x-rays [44]. Dosages for spine x-rays were reduced to between 1/6 and 1/9 of the standard dose, while delivering images that could be measured as accurately as standard radiographs. This technology is most useful when actual radiographs must be taken to look at the morphology of the vertebral column and an estimation of the spinal shape using non-radiographic methods is insufficient. The ability to take these images with the patient standing and subject to gravity is also a benefit.

## The use of MRI in imaging spinal deformity

Jaeger et al. [45] published their study on the use of MRI for measuring deformity in juvenile scoliosis as an alternative to radiographic follow-up. Schmitz et al. [46], representing the same research team, concentrated on the technique of image reconstruction by using a path through the centers of all intervertebral discs; the authors claimed the MRI could help in detecting scoliosis progression. Another publication by the same team reported on the possibility of assessing the sagittal plane deformity in the brace [47]. The authors emphasized that using the MRI for sagittal plane assessment is advantageous to avoid the lateral spinal radiography that presents increased entrance surface radiation dose comparing to anteroposterior or posteroanterior projections. As opposed to the upright physiological position of the human spine during radiography, the MRI examination was always executed in standard supine position, with the pelvis horizontal, the lower limbs straight, and the head flat.

An attempt to overcome the supine position-related problems resulted in an MRI study by Wessberg et al. [48], who investigated the deformity of the spine in a supine axially loaded position. A special axial loading device allowed for loading 15% to 20% of body weight on each foot.

An axially loaded MRI of patients with idiopathic scoliosis was used to study the mechanics of spinal deformity and demonstrated increase in Cobb angle but not in vertebral axial rotation under the load of 50% of body weight [49]. Technical notes were recently published regarding how to measure the Cobb angle on MRI and CT if both end vertebrae (proximal and distal) cannot be seen on one image [50].

When reviewing publications on the use of MRI for assessing spine deformity in idiopathic scoliosis, three aspects are observed: (1) the substitution of radiographic imaging with non-radiating techniques, (2) technical problems in drawing Cobb angles on MRI, and (3) attempting to simulate gravity conditions by applying axial loading.

It is important to note that the MRI technique, while able to assess the spine in 3D, did not define new parameters describing deformity in idiopathic scoliosis. Another category of publications concerning the application of MRI to evaluate deformity in idiopathic scoliosis relies on potentially assessing the functional aspects of the disease.

Chu et al. [51] studied the length of the vertebral column and the length of the spinal cord and demonstrated relative segmental lengthening of the vertebral column at the thoracic level in patients with severe thoracic scoliosis.

Kotani et al. [52] reported on chest wall and diaphragmatic movement using dynamic breathing MRI. Chu et al. studied the capabilities of MRI to assess lung volume, chest wall, and diaphragm motion [51] as well as lung function before and after spinal fusion [53].

## The frequency of x-rays necessary in adolescent idiopathic scoliosis (AIS) surveillance

Very little literature is published to indicate how often x-rays are necessary during scoliosis surveillance. The 2007 SOSORT Consensus paper on school screening recommended that children with an increased scoliometer reading be sent for an orthopaedic and radiographic evaluation [54]. According to this recommendation, it was suggested that children be x-rayed in two projections (AP and lateral) during the initial evaluation and then no more than once a year thereafter if curve progression and intervention are not indicated. Follow-up radiographs should be taken using the fewest projections possible, meaning that the AP only, and not the lateral view, should be taken, if possible. These recommendations were based on clinical consensus and not on scientific evidence.

In 2007, the American Academy of Orthopaedic Surgeons also published an opinion statement on school screening for scoliosis [55], which stated that children should be screened at ages 10 and 12, and that positive screenings should result in an orthopaedic consultation and, sometimes, an x-ray. No recommendation on the number of x-rays is given, but it is recommended that physicians avoid inappropriate use of x-rays by limiting exposure.

Italian guidelines written by the Italian Scoliosis Society [56] recommended that an x-ray in two projections be taken as part of the initial evaluation for scoliosis, with no more than one follow-up x-ray per year after that, except in cases of medical necessity. The 2007 SOSORT Consensus Report [54] stated that scoliosis experts agreed that x-rays should be performed at the time of first evaluation and then every 6–12 months afterward in an effort to limit the total number of x-rays. Experts also agreed that an in-brace (INB) x-ray was appropriate at the time a brace was prescribed.

## The use of x-rays in assessing brace effectiveness

The necessity and frequency of x-ray exposure in monitoring scoliosis concerns many parents in light of evidence that cumulative radiation exposure from x-rays increases a patient’s cancer risk. Never the less, the often-quoted adage that “a picture is worth a thousand words” is particularly true with scoliosis. In the era of evidence-based medicine, radiographic evaluation of scoliosis continues to be the most expedient, cost effective, and reliable assessment method. Historically, it has been the standard for determining Cobb angle, curve pattern, apices, end points, rotation, vertebral body shape, and structural anomalies. It is a vital tool in making clinical decisions.

In 1970, Dr. John H. Moe [57] described the essential components of successful bracing: cooperation of patient and parents, a properly constructed brace, and a knowledgeable orthopedic surgeon that closely monitors treatment. These elements are still pertinent today. To meet these criteria, we need to define a well-constructed brace and what it means to monitor treatment. An orthosis that optimally reduces the curve, improves spine balance, and does not cause discomfort is considered well-constructed. Monitoring includes not only comfort and compliance in the orthosis, but its effectiveness in reducing the curve while being worn. Watts et al. repeated this recommendation in their 1974 article [58], stating, “Critical analysis of a curve requires an adequate x-ray.” The initial pre-brace x-ray is the tangible “evidence” of the patient’s scoliosis and establishes a baseline against which to compare future x-rays. It also provides the equivalent of a blueprint that the orthotist uses to design and construct a proper orthosis. Watts also stated that the orthotist will do a better job if he has access to the INB x-ray. This novel concept in the early 70s resulted in the development of clinical teams, each member offering expertise in their specialty. As part of the treatment team, orthotists should see pre-brace x-rays and all subsequent INB and out-of-brace (OOB) films throughout the treatment period. The physician and orthotist should evaluate x-rays taken in brace to determine proper orthosis fit, curve correction, and central sacral spine balance [59].

Initial pre-treatment films serve as a baseline for comparing all future x-rays in or out of the brace and at the completion of treatment. Several studies showing successful results note that maximizing the initial INB Cobb angle correction is a predictor of success [58,60,61]. Also, the INB x-ray allows for a critical analysis of the brace design. By studying the INB x-ray, specific adjustments can be made to enhance correction. A thorough clinical evaluation of the patient in conjunction with a radiographic evaluation provides essential information for the orthotist constructing the orthosis and monitoring its effectiveness.

Most commonly, a standing PA x-ray taken after the thoraco-lumbar-sacral orthosis (TLSO) fitting reveals whether or not it has achieved the desired effect of reducing the curve(s) and re-establishing proper coronal plane balance However, no standard protocol for the timing of this xray exists. Some physicians/intuitions routinely take the INB x-ray the day of the orthosis fitting, while others suggest taking it two to four weeks following the brace fitting. (This facilitates viscoelastic changes through incremental loading of correctional forces).

Similarly, there is highly variable protocol regarding the timing of subsequent x-rays taken at 3-, 4-, or 6-month intervals, and equally variable protocol regarding whether these x-rays are taken in or out of the brace. Some physicians take every x-ray in the brace; some take an INB x-ray and OOB x-ray on the same day. Some alternate, taking an INB x-ray at one appointment, then an OOB x-ray at the next. Some institutions take x-rays while the patient is in the plaster mold prior to making the TLSO and then again after fitting the TLSO [62]. Others routinely take supine side bending films. Some take follow-up films every three to four months, regardless of a patient’s age, curve magnitude, or risk for progression, while others tailor a follow-up protocol to each patient while making a conscious effort to minimize radiation exposure.

Creating chest wall and/or sagittal plane deformities is a real possibility, as many TLSOs apply corrective forces to the torso via the ribs and soft tissue. It is also possible to change the curve patterns and magnitudes. Accurately assessing this through periodic OOB radiographs is critical. Changes in curve pattern or magnitudes necessitate corresponding changes in the design and function of the orthosis. This is of particular concern in treating the hyper mobile and malleable infantile/juvenile scoliosis patient.

The universal desire is to minimize the amount of x-ray exposure; however, the x-ray is an important diagnostic and monitoring tool essential for assessing the need for bracing and for monitoring its effectiveness. Clinical team members need periodic INB and OOB x-rays, to evaluate progress and make informed clinical decisions.

## Overall strategies for reducing x-ray exposure in pediatric deformity patients

In summarizing this literature review, evidence supports the following strategies:

• Recognize that scoliosis x-rays in a juvenile or adolescent population increase the risk of future malignancy, and employ methods to reduce their frequency.

• Methods that use no radiation should be used as part of a scoliosis evaluation. These can be low-tech, such as the Bunnell Scoliometer, or high-tech, such as surface topography scanners.

• When available, methods that use ultra-low-dose radiation should be used instead of standard radiographs.

• The MRI may have a place in the evaluation of scoliosis curves if methods are used to reproduce the gravitational forces present when standing.

• When radiographs are needed, methods that reduce exposure to sensitive tissues should be employed. Evidence for PA versus AP trunk radiographs is supported.

• Radiographic evaluation after brace fitting may still be an important part of ensuring proper curve correction.

## SOSORT 2012 consensus statements

Delphi Process: After the literature review was complete, the SOSORT Consensus committee developed a survey to determine how members were treating patients in an effort to reduce x-ray exposure. Using the Delphi Method, two rounds of surveys were distributed to identify statements of consensus. During each round of surveys, the statements were revised and clarified to more carefully represent the opinions of the group.

Because treatment principles are based on patient age and level of maturity, the following groups of patients were created to help ensure more precise consensus statements:

• 0–5 years of age (Congenital Scoliosis)

• 6–12 years of age (Early Onset Scoliosis)

• 13–18 years of age (AIS) Risser 0–1 Immature

• 13–18 years of age (AIS) Risser 2–3 Maturing

• 13–18 years of age (AIS) Risser 4–5 Mature

• 19–30 years of age (Post-AIS Surveillance)

When the SOSORT committee arrived at a final group of consensus statements, the statements were presented to the SOSORT membership at the Annual Meeting in Milan, Italy, for a vote. Voting members were able to vote for the statements as written, provide ideas to modify or clarify the statements, or reject individual statements that did not reflect their own practice. After evaluation of the votes and final modification of the statements, the following recommendations can be made:

### Consensus statements

#### Statement 1

A baseline x-ray of a new patient does not need to be taken to evaluate scoliosis if other clinical observations (i.e., scoliometer and physical examination) are normal.

#### Statement 2

For a patient with scoliosis, physician visits for clinical evaluation should be scheduled at the following intervals:

• For patients 0–5 years of age with congenital scoliosis: every 3 months

• For patients 6–12 years of age with early onset scoliosis: every 4 months

• For patients 13–18 years of age with AIS, Risser Stage 0–1: every 3 months

• For patients 13–18 years of age with AIS, Risser Stage 2–3: every 4 months

• For patients 13–18 years of age with AIS, Risser Stage 4–5: every 6 months

• For patients 19–30 years of age with AIS, Post-growth surveillance: every 24 months

#### Statement 3

For a patient with scoliosis, spinal radiographs should be scheduled at the following intervals:

• For patients 0–5 years of age with early onset scoliosis: every 6 months

• For patients 6–12 years of age with juvenile scoliosis: every 6 months

• For patients 13–18 years of age with AIS, Risser Stage 0–1: every 12 months

• For patients 13–18 years of age with AIS, Risser Stage 2–3: every 12 months

• For patients 13–18 years of age with AIS, Risser Stage 4–5: every 18 months

• For patients 19–30 years of age with AIS, Post-growth surveillance: every 24 months

#### Statement 4

A change in scoliometer reading and/or a change in appearance of trunk asymmetry should be the objective observations that trigger a scoliosis patient to receive a new radiograph.

#### Statement 5

When evaluating a patient with scoliosis, it is recommended that a PA, rather than AP, radiograph be taken to reduce the dose of radiation to breast tissue.

#### Statement 6

It is recommended that a lateral radiograph be taken during the first assessment of a scoliosis patient and not during every subsequent AP or PA radiograph, unless the patient has a significant sagittal plane deformity that appears to be changing.

#### Statement 7

CT scans may be useful to the surgeon for pre-operative evaluation of the scoliosis patient, but should not be routinely used for deformity evaluation.

#### Statement 8

MRI scans can be useful in the evaluation of neuro-anatomy in the scoliosis patient with a suspected neurological condition.

#### Statement 9

Non-radiographic modalities, such as physical examination, scoliometer readings, and surface topography, should be used first to detect curve progression in scoliosis patients.

#### Statement 10

When physical examination, scoliometer readings, and surface topography are used appropriately in the follow-up evaluation of the scoliosis patient, the number of subsequent radiographs can be reduced.

## Competing interests

The authors of this manuscript report that they have no competing interests to declare.

## Authors’ contributions

PK, EP, MC, JCdM, CR, TK, FZ, JW, LS, JB-S, TBG, JD, SN, JPO all contributed to by drafting and editing parts of the manuscript; PK, EP, MC, and LS also helped edit; PK, JCdM, CR, TK, FZ, JW, LS, JB-S, TBG, JD, SN, JPO, TM, and MR contributed by drafting the consensus statements. All authors read and approved the final manuscript.
